# Encephalitis temporally associated with live attenuated Japanese encephalitis vaccine: four case reports

**DOI:** 10.1186/1471-2334-11-344

**Published:** 2011-12-14

**Authors:** Na Jia, Qiu-Min Zhao, Xiao-Fang Guo, Jing-Xia Cheng, Chao Wu, Shu-Qing Zuo, Pei-Fang Dai, Jun-Ying Zhao, Jiu-Song Zhang

**Affiliations:** 1 State Key Laboratory of Pathogen and Biosecurity, Beijing Institute of Microbiology and Epidemiology, 20 Dong Da-Street, Fengtai District, Beijing 100071, People's Republic of China; 2 Yunnan Institute of Parasitic Diseases, Pu'er 665000, People's Republic of China; 3 Shanxi Center for Disease Control and Prevention, Taiyuan 030012, People's Republic of China

## Abstract

**Background:**

Japanese encephalitis (JE) vaccination is the most effective measure for preventing JE disease. The live attenuated JE vaccine, which has shown good efficacy and safety, has been widely used in China.

**Case presentations:**

We report four laboratory-confirmed JE cases detected in JE-endemic areas during the JE virus (JEV) transmission season, who all received a first dose of live attenuated JE vaccine within 2 weeks prior to the onset of illness. All cases presented with acute encephalitis and rapidly reduced consciousness. All cerebrospinal fluid (CSF) samples from the patients were positive for JEV-specific immunoglobulin M (IgM) antibodies, but viral isolation and polymerase chain reaction (PCR) detection of JEV were both negative.

**Conclusions:**

It is difficult to identify a causal link between the disease and the vaccination, as the source of positive CSF JEV IgM antibodies might be natural JEV infection or possibly due to a traumatic lumbar puncture. Our observations highlight the need for public health officers and doctors to consider reasonable vaccination policies during the JE season. In addition, continued surveillance as well as thorough investigation of any events that occur after JE vaccination is necessary.

## Background

Japanese encephalitis (JE), a major cause of acute viral encephalitis, affects approximately 30,000-50,000 of the population and causes up to 15,000 deaths annually in Asia. The disease occurs throughout most of Asia and parts of the western Pacific. In parts of Asia, it is principally a disease of children living in rural areas [1]. In the absence of specific antiviral treatment, the World Health Organization (WHO) and most affected countries have placed a high public health priority on vaccination.

After a nationwide vaccination program was initiated in the late 1970s in China, the incidence of JE dramatically decreased from 20.92/100,000 people in 1971 to 0.23/100,000 people in 2008 [1]. In China, inactivated (P3 strain) and live attenuated (SA14-14-2 strain) JE vaccines were developed. The inactivated vaccine was introduced in the mid-1970s and played a major role in reducing JE incidence in the 1980s and 1990s [1]. The live attenuated vaccine, developed in 1988, provided excellent effectiveness (88%-96%) and good safety [2-7] and replaced the inactivated vaccine in many provinces. At present, more than 110 million doses of JE vaccines are produced annually in China, in excess of domestic demand, and nearly 120 million children have been reached through vaccine campaigns and routine immunization [1]. Recently, one report summarized a small number of adverse events temporally associated with JE vaccination in China from 1994 to 2004. The majority of these cases were detected in county or lower level hospitals and were characterized by hypersensitivity-type reactions that occurred shortly after vaccination [8]. However, this report did not clearly discriminate the type of vaccine administered. Here, we describe four laboratory-confirmed JE infections temporally associated with live attenuated JE vaccine administration.

JE cases were detected through viral encephalitis surveillance in two JE-endemic rural districts in Yunnan, China, from May to December 2009. The two districts showed a high JE annual average incidence of 2.47-4.4/100,000 people from 2000 to 2007, and JE cases occur during every month of the year, but peak from July to October [9]. JE was confirmed by one or more of the following: a JE virus (JEV)-specific immunoglobulin M (IgM) antibody-capture enzyme-linked immunosorbent assay (MAC-ELISA) (Shanghai B & C Enterprise Development Co. Ltd, Shanghai, China) [10], reverse transcription semi-nested polymerase chain reaction (PCR), or virus isolation from serum or cerebrospinal fluid (CSF) obtained from patients with viral encephalitis who were admitted to local county hospitals. The JEV MAC-ELISA remains the principal method for laboratory confirmation of JE in China [10]. JEV ELISA for the patients was repeated both by the local hospital and by Beijing Institute of Microbiology and Epidemiology. Of the 84 patients investigated, 36 (43%) were positive for JEV infection. Three JEV isolates were obtained (GenBank no. GU371870; GU371871; GU371872) and all were identified as genotype I. The viral encephalitis surveillance was performed after consultation with the patients or their guardians and after the receipt of written consent. The study-related information was used anonymously. The Institutional Review Board of the Beijing Institute of Microbiology and Epidemiology approved the research involving human materials.

## Case presentations

Of 36 laboratory-confirmed JE cases, four cases had been vaccinated with a first dose (0.5 ml) of live attenuated JE vaccine (Chengdu Institute of Biological Products, China) within 2 weeks prior to the onset of illness. Characteristics of the cases are summarized in Table 1. **Case 1**, a nine-month-old girl, was admitted to local pediatric hospital A on 4 September with rapidly progressive encephalopathy. She was febrile on 31 August. At the admission, she had a temperature of 38.3°C, and was drowsy. Neck stiffness, positive Kernig's sign, uncontrolled shaking of the body and hypertonia were also present. No rash was noticed. Eleven days prior to the onset of fever, she received the live attenuated JE vaccine. **Case 2 **was a previously healthy four-year-old boy. He was admitted to local hospital B on 30 June with a severe headache. At hospital admission, he had a temperature of 39°C, vomiting, neck stiffness and positive Kernig's sign, uncontrolled shaking of the body, and ultimately slipped into a coma. He had been administered the JE vaccine 1 week prior to his illness. **Case 3 **was a previously healthy four-year-old girl. She was admitted to hospital C on 1 July with symptoms of fever, severe headache and altered mental status. On admission, the patient's temperature was 37°C, and this rose to 40°C after hospitalization. No vomiting or diarrhea was noted. Severe meningeal irritation signs were also present. She then developed abnormal muscle tone, and finally became unconscious. She had received the JE vaccine 14 days prior to the onset of illness. **Case 4**, a five-year-old boy exhibiting normal growth, was febrile on 7 July and was admitted to hospital C on 10 July with fever of 40°C. The prodromal symptoms of the boy included headache, nausea and vomiting. Irritability and neck stiffness were also present but the patient was alert. During a physical examination, abnormal muscle tone (hypertonia) and abnormal limb reflexes (hyperreflexia) were detected. He then rapidly lost consciousness. 11 days prior to the onset of fever, he received one dose of the JE vaccine. No allergic reaction or other remarkable medical history was recorded in the four cases. Table 1 illustrates the interval between illness onset and CSF with or without serum sample collection (≤ 4 days).

**Table 1:** Characteristics of the four cases in this study

Characteristics	Case 1	Case 2	Case 3	Case 4
Age	9 months	4 years	4 years	5 years

Sex	Female	Male	Female	Male

Days from vaccination to onset of disease	11	7	14	11

Past medical history	None	None	None	None

Other vaccines	None	None	None	None

Days from the onset of symptoms to CSF with or without serum sample collection	4	1	4	3

CSF color	Clear	Clear	Clear	Clear

JEV IgM antibodies in CSF*	Positive	Positive	Positive	Positive

JEV IgM antibodies in serum*	Positive	NA	Positive	NA

* Serological testing was conducted by Japanese encephalitis virus-specific immunoglobulin M (IgM) antibody-capture enzyme-linked immunosorbent assay

CSF: cerebrospinal fluid

NA: not available

Laboratory tests for **Case 1 **indicated a normal serum leukocyte count (8.5 × 10^3 ^cells/mm^3^), high levels of neutrophilic granulocytes (74.4%) and a normal CSF leukocyte count (8 WBCs/mm^3^; normal range: 0-10 WBCs/mm^3^). Elevated protein levels (560 mg/L; normal range: 200-400 mg/L) but normal glucose levels (3.31 mmol/L; normal range: 2.8-4.4 mmol/L) were detected in the CSF. A Gram stain revealed no organisms. The CSF biochemical parameters of other three cases were not available. All CSF samples were positive for JEV-specific IgM antibodies as analyzed by the MAC-ELISA (Table 1). Viral isolation and PCR for JEV were both negative for the CSF and serum samples. Testings for other aetiologies of meningoencephalitis were not conducted. Brain magnetic resonance imaging (MRI) was not performed in these hospitals.

The therapy for all cases consisted of supportive care and the management of complications, such as the use of empirical intravenous antibiotics to prevent possible bacterial infection and the injection of intracranial tension-lowering agents (e.g., mannitol). All patients were discharged from the hospital, but their sequelae were not followed-up by interview.

## Discussion

In the present study, we report four laboratory-confirmed JE cases, confirmed by positive IgM levels in their CSFs, who all received a first dose of a live attenuated JE vaccine within 2 weeks prior to the onset of symptoms. It is difficult to identify a causal link between the disease and JE vaccination, because no etiological evidence such as positive viral isolate or nucleotide was found.

It might be feasible to consider the events might be due to JE vaccination. However, the live SA 14-14-2 attenuated vaccine is safe based on available data. A large randomized clinical trial involving 26,239 children demonstrated good short-term safety of the live attenuated vaccine. No cases of encephalitis or meningitis occurred in subjects who received the vaccine [4]. In a study begun in 1985, 1026 children were vaccinated with live-attenuated vaccine (SA14-14-2 strain), and none of these children had temperatures greater than 37.4°C or other systemic reactions during the observation period after vaccination [5]. Until now, there have been no other reported cases of neurological adverse events despite the live attenuated vaccine being used in several hundred million children over a period of 20 years [11]. During our further interview, the batch numbers of the vaccine administrated by the four cases were not clearly remembered by their guardians. It made our in-depth investigation of the vaccine safety difficult.

Furthermore, it is important to note that all cases were detected during the peak circulation period of JEV in a JE-endemic area with relatively high JE incidence. In this study, 36 (43%) out of 84 children with viral encephalitis were diagnosed with JE. This high rate of JEV transmission suggests a high risk of natural infection. The incubation period for JE is 5-15 days [7]. Therefore, it may also suggest a reasonable likelihood of these cases being caused by a wild-type virus. JE vaccination is very unlikely to prevent a wild-type infection acquired around the same time as vaccination was given.

A third possibility, although less likely, is a traumatic tap when lumbar puncture was conducted with neurological infection due to another etiology. Anti-JEV IgM would be expected in the serum of patients vaccinated with live attenuated JE vaccine, and indeed was detected in two cases reported. In the case of a traumatic lumbar puncture, the anti-JEV IgM detected in CSF may be from blood contamination.

## Conclusions

This report highlights the need for public health officers and doctors to consider reasonable vaccination policies during the JE season. JE vaccination should be recommended outside of a well-defined JEV transmission season. If vaccination is administered during the transmission season, a clear explanation should be provided that the vaccine is not likely to prevent illness if a person has already been infected at the time of vaccination. In addition, continued good quality surveillance as well as thorough investigation of any events that occur after JE vaccination is necessary.

## Abbreviations

JE: Japanese encephalitis; JEV: Japanese encephalitis virus; ELISA: Enzyme-linked immunosorbent assay.

## Competing interests

The authors declare that they have no competing interests.

## Authors' contributions

J-SZ conceived the study, participated in its design and was involved in the drafting of the manuscript, revising it critically for important intellectual content and approving the final version. NJ participated in the data analysis and helped to draft the manuscript. Q-MZ and S-QZ conducted the laboratory testing. J-YZ, P-FD, CW and X-FG coordinated the sample collection. All authors read and approved the final manuscript.

## Consent

Written informed consent was obtained from the parents of the patients for publication of this case report. A copy of the written consent is available for review by the Editor-in-Chief of this journal.

## Pre-publication history

The pre-publication history for this paper can be accessed here:

http://www.biomedcentral.com/1471-2334/11/344/prepub
